# Morphometric characterization of microglial phenotypes in human cerebral cortex

**DOI:** 10.1186/1742-2094-11-12

**Published:** 2014-01-21

**Authors:** Susana G Torres-Platas, Samuel Comeau, Adeline Rachalski, Gregory Dal Bo, Cristiana Cruceanu, Gustavo Turecki, Bruno Giros, Naguib Mechawar

**Affiliations:** 1 McGill Group for Suicide Studies, 6875 LaSalle Blvd, Verdun, Québec H4H 1R3, Canada; 2 Douglas Mental Health University Institute, 6875 LaSalle Blvd, Verdun, Québec H4H 1R3, Canada; 3 Department of Neurology and Neurosurgery, McGill University, Montreal, Canada; 4 Department of Human Genetics, McGill University, Montreal, Canada; 5 Department of Psychiatry, McGill University, Montreal, Canada

**Keywords:** Human, microglia, morphology, IBA1, anterior cingulate cortex

## Abstract

**Background:**

Microglia can adopt different morphologies, ranging from a highly ramified to an amoeboid-like phenotype. Although morphological properties of microglia have been described in rodents, little is known about their fine features in humans. The aim of this study was to characterize the morphometric properties of human microglia in gray and white matter of dorsal anterior cingulate cortex (dACC), a region implicated in behavioral adaptation to neuroinflammation. These properties were compared to those of murine microglia in order to gain a better appreciation of the differences displayed by these cells across species.

**Methods:**

Postmortem dACC samples were analyzed from 11 individuals having died suddenly without any history of neuroinflammatory, neurodegenerative, nor psychiatric illness. Tissues were sectioned and immunostained for the macrophage marker Ionized calcium binding adaptor molecule 1 (IBA1). Randomly selected IBA1-immunoreactive (IBA1-IR) cells displaying features corresponding to commonly accepted microglial phenotypes (ramified, primed, reactive, amoeboid) were reconstructed in 3D and all aspects of their morphologies quantified using the *Neurolucida* software. The relative abundance of each morphological phenotype was also assessed. Furthermore, adult mouse brains were similarly immunostained, and IBA1-IR cells in cingulate cortex were compared to those scrutinized in human dACC.

**Results:**

In human cortical gray and white matter, all microglial phenotypes were observed in significant proportions. Compared to ramified, primed microglia presented an average 2.5 fold increase in cell body size, with almost no differences in branching patterns. When compared to the primed microglia, which projected an average of six primary processes, the reactive and amoeboid phenotypes displayed fewer processes and branching points, or no processes at all. In contrast, the majority of microglial cells in adult mouse cortex were highly ramified. This was also the case following a postmortem interval of 43 hours. Interestingly, the morphology of ramified microglia was strikingly similar between species.

**Conclusions:**

This study provides fundamental information on the morphological features of microglia in the normal adult human cerebral cortex. These morphometric data will be useful for future studies of microglial morphology in various illnesses. Furthermore, this first direct comparison of human and mouse microglia reveals that these brain cells are morphologically similar across species, suggesting highly conserved functions.

## Introduction

Microglia have traditionally been recognized as the innate immune cells mediating inflammatory responses in the central nervous system (CNS). In recent years, however, it has become increasingly clear that ramified “resting” microglia also participate actively in fundamental aspects of neuronal activity, including structural and functional plasticity [1-3]. Ramified microglia can respond to subtle microenvironmental changes arising from a wide variety of factors such as pathogens [4-6], stress [7,8], and injury, [9-11] with what is commonly referred to as microglial activation. As mainly described in rodent studies of CNS injuries, this involves a rapid alteration of cell metabolism and function [12-14], which can be accompanied by a graded spectrum of morphological changes that transform highly ramified microglia into amoeboid-phagocytic microglia [12,14-16]. Following cell activation, highly branched microglia can reabsorb stochastically (and reversibly) into the cell body before transitioning to a dynamic motility stage with cycles of extension and retraction of new processes. Fully activated microglia then initiate a locomotor stage, by which they migrate throughout the tissue [14,17]. Ramified microglia can also reach intermediate phenotypes before returning to a ramified morphology, without ever becoming amoeboid-like. Along the complete activation sequence described above, four major phenotypes are usually distinguished in rodents based on distinct morphological [14,15] and molecular criteria [18-21]: ramified, primed, reactive, and amoeboid.

Analyzing microglial morphology and function in human brains is obviously more challenging. Postmortem studies have confirmed the existence of various morphological phenotypes [13,22,23] that had been previously described in rodents. Furthermore, some of the morphological and molecular mechanisms underlying human microglial reactivity have been described during development, [24-26], as well as in pathological conditions such as Creutzfeldt-Jakob [27,28], Parkinson’s disease [29-31] Alzheimer’s disease [22,32,33] and multiple sclerosis [34-36]. Despite these advances, there is a current lack of detailed knowledge on the fine properties of microglia in the human brain, and how these properties may generally compare to those of rodents, which are commonly used as models in biomedical research. Here, we report the first comprehensive morphometric analysis of the different microglial phenotypes found in postmortem samples of dorsal anterior cingulate cortex (dACC), an area that has been associated with the behavioral response to neuroinflammation [37], from adult individuals having died with no history of inflammatory, neurological or psychiatric illness. Tissue sections were immunostained for ionized calcium binding adapter molecule 1 (IBA1), a calcium-binding protein specifically expressed in macrophages and microglial cells [38], and the morphological features (cell-body shape and size, length and branching of processes) of randomly selected IBA1-immunoreactive cells corresponding to each major morphological phenotype were measured following their 3-dimensional reconstruction. In addition, similar analyses were carried out in mouse cortex for comparative purposes. This study not only provides fundamental information on the fine characteristics of human microglia, but also highlights the morphological similarities between human and mouse cortical microglia. Altogether, we propose measurable criteria for the differentiation of human microglial phenotypes that could be applied in future postmortem studies of pathological conditions.

## Materials and methods

### Human and mouse brain tissues

This study was approved by the Douglas Hospital Research Ethics Board, and written informed consent from next-of-kin was obtained for each subject. Fresh-frozen postmortem brain samples from the right hemisphere of individuals having died accidentally without any psychiatric, neurological, or inflammatory illnesses (n = 11) were provided by the Douglas-Bell Canada Brain Bank. The average age at death of these subjects (10 male and 1 female) was 48 ± 5.2 years old. The average postmortem interval (PMI) was 57.5 ± 5.4 hours, the interval between death and storage of the body at 4˚C (refrigeration delay) was 5.3 ± 2.0 hours, and the average brain pH was 6.6 ± 0.08. All subjects had died suddenly, without agony, from cardiovascular conditions (n = 6), road accidents (n = 2), or intoxication (n = 3). Brain samples were dissected from the dACC, adjacent to the dorsal part of the genu of the corpus callosum (BA24) [39,40], as described previously [41]. After fixation by immersion in 10% formalin, tissue blocks were cut into serial 50-μm-thick coronal sections using a freezing microtome. Every 12th section collected was processed for free-floating IBA1 immunohistochemistry as detailed below.

Adult male C57Bl6 mice (1.5 to 3.0 months old; n = 4) were used in this study. All procedures were approved by the Douglas and McGill Animal Care Committees. Three mice were deeply anesthetized with a solution containing ketamine and xylazine (0.1 mg/g, intraperioneal) and perfused intra-cardially with 4% paraformaldehyde (PFA) in 0.1 M PBS, pH 7.4, 50 ml per mouse. Brains were removed, post-fixed overnight by immersion in the PFA solution at 4°C, and washed in PBS. One mouse was used to evaluate the effects of PMI on IBA1-IR cell distribution and morphology. This animal was sacrificed by cervical dislocation and kept 11 hours at room temperature before being placed for 32 h at 4°C, to mimic human PMI conditions. The brain was then dissected and fixed by immersion in a 4% PFA solution for 48 hours at 4°C. All mouse brains were cut with a vibrating microtome into 50-μm coronal sections (brain slices corresponding to plates from +2.34 mm to -0.46 mm) containing cingulate cortex [42], and kept in PBS until further use. Samples were washed with PBS and processed for free-floating IBA1 immunohistochemistry as detailed below.

### IBA1 immunohistochemistry

**Figure 1:**
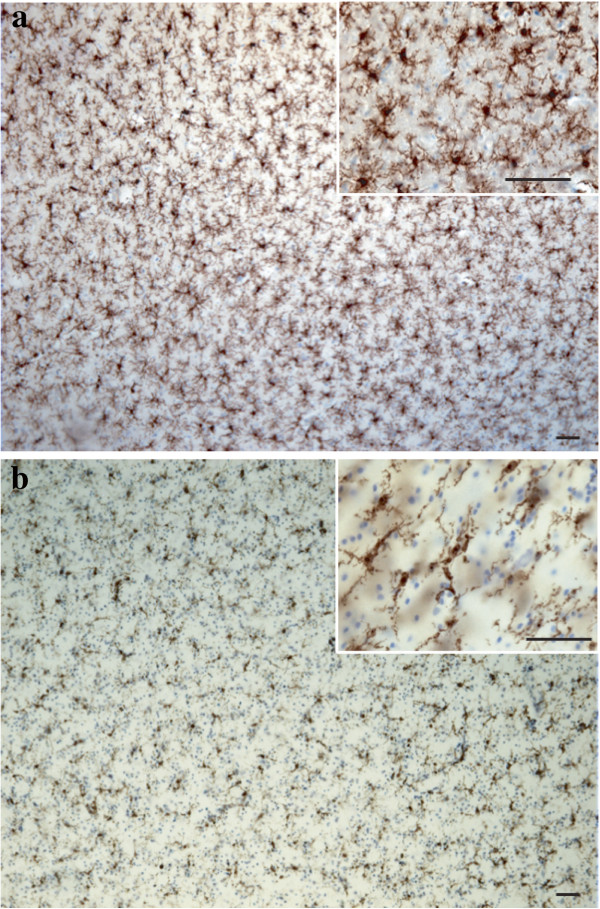
**Distribution of ionized calcium binding adaptor molecule 1 (IBA1)-immunoreactive (IR) cells in the gray and white matter human dorsal anterior cingulate cortex (dACC) counterstained with cresyl violet.** Human microglial cells in the dACC appear evenly distributed across the cortical layer in the gray matter **(a)** and aligned to myelinated tracts in the white matter **(b)**. Scale bars: 50 μm.

All incubations occurred at room temperature. Prior to immunohistochemical labeling, human tissues underwent antigen retrieval by incubating sections for 10 minutes in a solution of Tris-buffered saline (TBS) containing 20 μg/ml proteinase K, followed by a 10-minute incubation in distilled water containing 3% H_2_O_2_. Sections were then pre-incubated for 24 h in TBS + 0.05% tween containing 2% normal goat serum, before being transferred for 48 hours into the same solution containing polyclonal rabbit anti-IBA1 (1:1000; WAKO Chemicals USA, Inc., Richmond, VA, USA). This was followed by 1 hour incubation in biotinylated goat anti-rabbit antibody (1:1,000; Vector Laboratories Inc., Burlington, ON, Canada), and the avidin-biotin complex procedure (ABC Kit, Vectastain Elite, Vector Laboratories Inc., Burlington, ON, Canada) for 30 minutes. Labeling was revealed with a diaminobenzidine kit (Vector Laboratories Inc., Burlington, ON, Canada) and samples were counterstained with cresyl violet to better differentiate gray and white matter (Figure 1). Sections were mounted on glass slides, dehydrated, and coverslipped with Permount (Fisher Scientific Inc., Pittsburgh, PA, USA). Mouse brain sections underwent the same procedures, with the exception of the antigen retrieval step.

### Morphometric analyses of microglial phenotypes in human dACC

**Figure 2:**
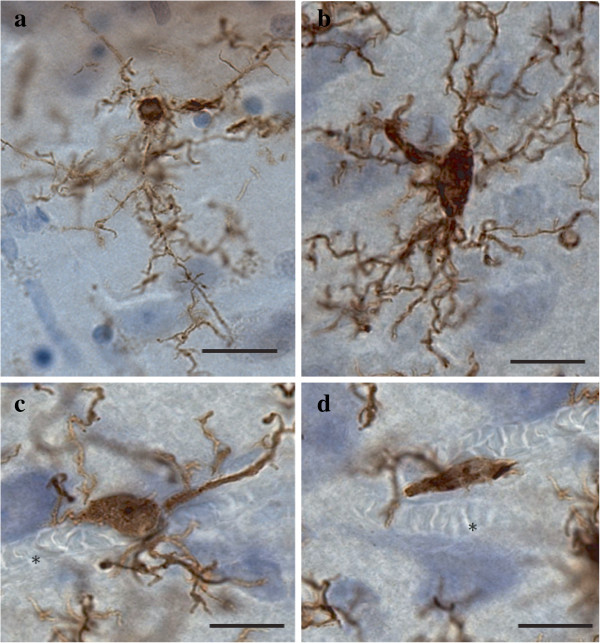
**Four main phenotypes represent the population of resident IBA1-IR cells in human dACC gray matter.** Ramified microglia **(a)** display a small circular cell body with highly ramified processes. Primed microglia **(b)** present a bigger and less round cell body with similar ramification patterns when compared to the ramified phenotype. Reactive microglia **(c)** display an amoeboid cell body but still present a few ramified processes compared to amoeboid microglia **(d)**, which can present, at most, two unramified processes or be completely devoid of them. These cells are occasionally observed to be associated with blood vessels (asterisks). Scale bars: 10 μm.

**Figure 3:**
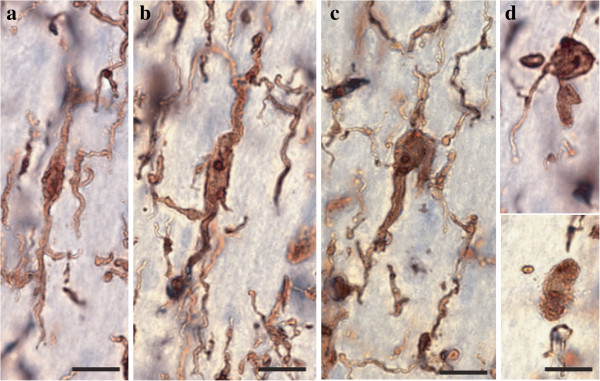
**Four main phenotypes represent the population of resident IBA1-IR cells in human dACC white matter.** Ramified microglial cell body and highly ramified processes appear aligned to white matter tracts **(a)**. Primed microglia display a wider cell body in the primed phenotype **(b)** compared to the ramified phenotype, but their processes and cell body retain a similar alignment. Reactive microglia present an amoeboid-shaped rounder cell body with a few ramified processes **(c)**, whereas amoeboid microglia display a characteristic amoeboid-shaped cell body extending one or two unramified processes (top panel) or are completely devoid of processes (bottom panel) **(d)**. Scale bars: 10 μm.

A general assessment of IBA1-immunoreactive (-IR) cells was first conducted to evaluate the relative distribution and abundance of microglial phenotypes in dACC gray and white matter. In all subjects, microglia in the gray matter were generally randomly distributed across and within layers, whereas they appeared to be aligned with myelinated fibers in the adjacent white matter (Figure 1). Four distinct morphological phenotypes were easily recognizable in both gray and white matter. These morphologies corresponded to the previously described microglial phenotypes classically associated with differing states of activation: ramified, primed, reactive, and amoeboid [12,14,15,22,43]. In human dACC, IBA1-IR cells were categorized using the following distinctive features: ramified microglia displayed a small but defined cell body that appeared spherical in the gray matter (Figure 2a) and ellipsoid in the white matter (Figure 3a). In both cortical compartments, ramified microglia displayed several highly branched processes. Primed microglia in gray matter remained highly ramified, albeit with fewer higher-order branches, but presented a distinctive ellipsoid-like soma (Figure 2b). In the white matter, primed microglia were also highly ramified, but displayed a noticeably wider cell body (Figure 3b). Reactive and amoeboid microglia both presented amoeboid-shaped cell bodies. The processes extended by reactive microglia were less extensive and generally longer than the cell body diameter (Figures 2c and 3c), whereas amoeboid microglia were either devoid of processes or had few unbranched processes seen to be within the length of the cell-body diameter (Figures 2d and 3d).

**Figure 4:**
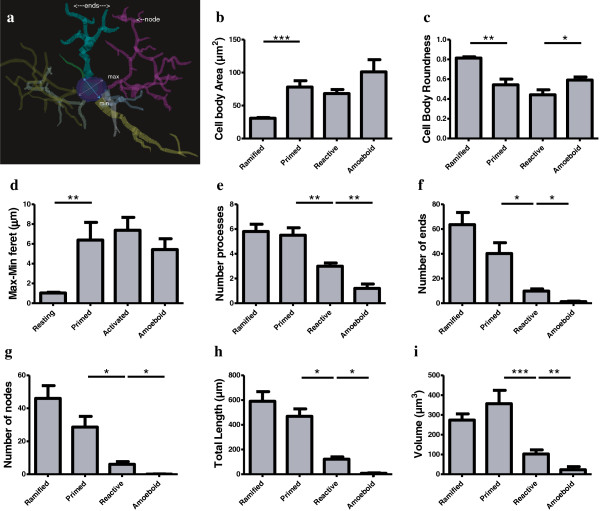
**Microglial phenotypes in human dACC gray matter are characterized by significant changes in the cell body and processes.** Primed microglia display a cell body of greater area **(a)** and of decreased roundness **(b)**, as reflected by a significantly increased difference between the maximum and minimum feret (max-min feret) **(c)** compared to the ramified phenotype. The cell body morphology of reactive microglia appears statistically similar in area **(a)**, roundness **(b)** and max-min feret **(c)** as primed microglia, but presents a decrease in roundness **(b)** when compared with amoeboid microglia. A reconstruction of a primed microglia shows the shortest (blue arrow; min) and longest chord (violet arrow, max) representing the maximum and minimum feret, respectively, and the ramification patterns represented by the ends and nodes of the processes **(d)**. Ramified and primed microglia project similar numbers of primary **(e)** and higher-order processes **(f-g)**. Reactive microglia display fewer first-order (and overall) branches **(e-g)**, as well as shorter total process length **(h)** and volume **(i)**. Amoeboid microglia have a significant decrease of primary and higher order branches **(f-g)**, as well as a significant decrease in total process length **(h)** and volume **(i)** compared to reactive microglia, *P <0.01, **P <0.001, ***P <0.0001.

Having performed a preliminary assessment that revealed very little intra-phenotypic morphological variability between subjects, we proceeded by analyzing the first 10 IBA-IR cells that corresponded unambiguously to the above-described features corresponding to each phenotype. We analyzed a total 40 gray matter and 40 white matter microglia, with 10 cells/phenotype being randomly selected and reconstructed across subjects. On average, 7.4 ± 1.0 cells per subject were traced, reconstructed, and analyzed. Cells were sampled throughout the cortical thickness, but since no noticeable difference was seen between layers, laminar distributions were not recorded. Cells were traced, reconstructed, and their morphometric features characterized as previously described [44]. In brief, under a 100× (Numerical aperture 1.4) oil immersion objective (Olympus BX51 light microscope, Olympus America Inc., Richmond Hill, On, Canada) processes were analyzed in three dimensions within single sections using a computer-based tracing system (*Neurolucida* v. 8.10.2, MBF Bioscience, Williston, VT, USA), whereas cell bodies were analyzed in two dimensions (area at its largest cross-sectional diameter). Cell body area, maximum and minimum feret diameter, roundness as well as number, length, branching points (nodes and ends) and volume of processes were measured for each IBA1-IR cell. A spherical cell body is calculated by the ratio between feret diameters. Feret is defined as the distance between two parallel lines drawn tangentially to the cell body; the minimum feret is the shortest chord drawn in the cell body and the maximum Feret is the longest, as shown in the blue and purple lines respectively in Figure 4a. In a spherical cell body, the difference between the maximum and minimum ferets (max-min feret) tends to zero.

### Quantitative assessment of microglial phenotype distribution in human dACC

To assess the proportions of the different microglial phenotypes present in gray and white matter, we conducted a quantitative analysis in dACC sections from five individuals, using a semi-unbiased stereological approach with an optical fractionator probe allowing for 3-dimensional quantification with the light microscope connected to a stereology workstation (Stereo Investigator; MBF Bioscience). This method estimates the total number of cells in a unit of tissue volume with an optical probe providing counts through the z-axis. The sampling process was performed by adding a grid of dimensions 3,137 μm by 2,651 over the section in the white matter. We examined a counting frame measuring 150 μm to approximately 250 μm with a 60× objective (NA 1.35). Consistent with the stereological methods of the dissector probe, we counted only cells with a cell body that fell within the counting frame and that did not contact the exclusion lines when they came into focus within a 15-μm-thick optical dissector. To avoid counting cells in non-representative areas of the tissue, we set top and bottom guard zones at 1 μm of the section thickness. To control for the variation tissue processing, the volume of each individual counting frame was calculated with the area of the frame multiplied by the thickness of each counting site. A total of 3,604 cells were counted (average of 720 ± 67 cells per subject). The relative proportion of each of the four morphological phenotypes was calculated, and the total number of cells of each phenotype was divided by the total volume of the counting sites.

### Morphometric analyses of microglial phenotypes in mouse cingulate cortex

In contrast to what was observed in human tissues, IBA1-IR cells present in cingulate cortex of young adult mice were overwhelmingly of the ramified phenotype. In order to gain a general quantitative appreciation of the morphometric differences and similarities between human and murine microglia, and given that virtually no variability was observed between animals, we reconstructed and analyzed cells in a single mouse. Thus, a total of 20 ramified cells (ten per cortical compartment) were randomly selected in the cingulate cortex of a 1.5 month-old mouse and analyzed as mentioned above.

### Statistical analyses

Statistical analyses were performed using PASW Statistics 18 (Statistical Product and Service Solutions, Chicago, IL, USA) and Prism 5 (GraphPad Software, Inc., La Jolla, CA, USA). All measurements were expressed as mean ± standard error of the mean (SEM), and *P* ≤0.05 was considered significant in all statistical tests. Normality was assessed using the Shapiro-Wilk test, and two-tailed *t*-tests were used for normally distributed data, with the Welch correction in case of significant difference of variances. *U*-tests were used for non-normally distributed data. A Bonferroni correction was performed for each dependent variable to counteract for type one error in multiple comparisons.

## Results

### Qualitative and quantitative features of microglial cells in human dACC

#### ***Gray matter***

The ramified, primed, reactive and amoeboid microglial phenotypes were consistently observed in the gray matter of all dACC samples. IBA1-IR cells with highly branched processes represented the majority of cells observed in this region. Our stereological estimates indicate that nearly 16% of IBA1-IR cells were of the ramified phenotype, while about 34% were of the primed phenotype. In general, all phenotypes were seen to be evenly distributed throughout cortical layers, with no overlapping domains (Figure 1a). However, the distribution and relative space between IBA-IR cells varied within and between subjects. Compared to the other phenotypes, ramified microglia displayed a characteristic small and spherical cell body extending a large number of primary and higher-order processes (Figure 2a). All other phenotypes were clearly distinct from ramified microglia in that they had an amoeboid-like cell body (Figure 2). Primed microglia projected similar numbers of primary and higher-order processes than ramified microglia (Figure 4e), but clearly displayed a cell body of greater area (*U*_(18)_ 0.0, *P* <0.0001, Figure 4b) and of decreased roundness (*t*_Welch (9)_ 4.612, *P* = 0.0013; Figure 4c). This was reflected by a significant increase in minimum feret (*t*_Welch (11)_ 6.46, *P* <0.0001) and max-min feret (*U*_(18)_ 0.0 *P* = 0.0002; Figure 4d) compared to the ramified phenotype. The reactive and amoeboid phenotypes in the gray matter represented 32% and 18% of the total number of IBA1-IR cells in dACC, respectively. Although the cell body morphology of reactive microglia, in comparison to primed microglia, was statistically similar in al measured parameters (area, roundness and max-min feret), the processes of reactive microglia displayed significantly fewer first-order (and overall) branches (*U*_(18)_ ≤9.50, *P* ≤0.0025, Figure 4e), as well as significantly shorter total process length (*t*_Welch (10)_ 5.50 *P* = 0.0003, Figure 4h) and volume (*U*_(18)_ 5.00, *P* = 0.0002, Figure 4i). Amoeboid microglia had an increase in cell body roundness (*t*_(18)_ 2.49, *P* = 0.022, Figure 4c), compared to reactive microglia, with no significant differences in area, minimum feret, maximum feret and max-min feret. What characterized this phenotype, however, was a significant decrease of primary (*U*_(18)_ 9.50, *P* = 0.0015, Figure 4e) and higher-order branches (*U*_(18)_ 0.0, *P* = 0.0002, Figure 4f & 4 g), as well as a significant decrease in total process length (*U*_(18)_ 0.0, *P* = 0.0001, Figure 4h) and volume (*U*_(18)_ 11.00, *P* = 0.0021, Figure 4i) compared to reactive microglia. On occasion, a few IBA1-IR amoeboid-like cells were observed in close proximity of larger blood vessels (Figure 2). All results for gray matter cells are summarized in Table 1.

**Table 1:** Quantitative features of IBA1-IR phenotypes in human dACC gray matter

**Cell body**					
	**Area (μm2)**	**Roundness**	**Min feret (μm)**	**Max feret (μm)**	**Max-min feret (μm)**
**Ramified**	30.8 ± 0.9	0.8 ± 0.0	5.9 ± 0.1	6.9 ± 0.1	1.0 ± 0.0
Range	26.7 to 36.0	0.7 to 0.8	5.3 to 6.4	6.5 to 7.6	0.8 to 1.2
**Primed**	79.1 ± 9.6	0.5 ± 0.0	8.1 ± 0.3	14.5 ± 1.9	6.3 ± 1.7
Range	52.4 to156.9	0.2 to 0.7	7.0 to 10.0	9.3 to 28.6	2.3 to18.5
**Reactive**	68.2 ± 6.1	0.4 ± 0.0	7.2 ± 0.5	14.6 ± 1.1	7.3 ± 1.3
Range	31. 8 to 95. 9	0.2 to 0.6	5.0 to 10.8	8.1 to 21.7	3.1 to 10.9
**Amoeboid**	101.0 ± 18.5	0.5 ± 0.0	9.1 ± 0.6	14.6 ±1.5	5.4 ± 1.0
Range	41.0 to 225.0	0.4 to 0.7	6.0 to 13.3	9.9 to 27.7	3.1 to 14.
**Processes**					
	**Primary processes**	**Ends**	**Nodes**	**Total length (μm)**	**Volume (μm**^**3**^**)**
**Ramified**	5.8 ± 0.5	63.6 ± 9.8	46 ± 7.7	590.6 ± 77.5	274.1 ± 31.2
Range	3.0 to 9.0	25 to 104.0	16.0 to 78.0	294.5 to 979.4	105.9 to 407.4
**Primed**	5.5 ± 0.6	40.2 ± 7.7	28.6 ± 6.5	496.0 ± 60.1	356.8 ± 67.4
Range	3.0 to 8.0	14 to 91.0	9.0 to 66.0	287.3 to 793.0	86.5 to 772.4
**Reactive**	3.0 ± 0.2	9.8 ± 1.8	6.1 ± 1.5	122.4 ± 18.6	102.6 ± 20.8
Range	2.0 to 5.0	5.0 to 20.0	2.0 to 15.0	50.0 to 215.9	36.6 to 224.3
**Amoeboid**	1.2 ± 0.3	1.3 ± 0.3	0.1 ± 0.1	8.5 ± 3.4	23.6 ± 15.1
Range	0.0 to 2.0	0.0 to 3.0	0.0 to 1.0	0.0 to 31.2	0.0 to 145.9

Results are presented as mean ± standard error of the mean and range.

#### ***White matter***

**Figure 5:**
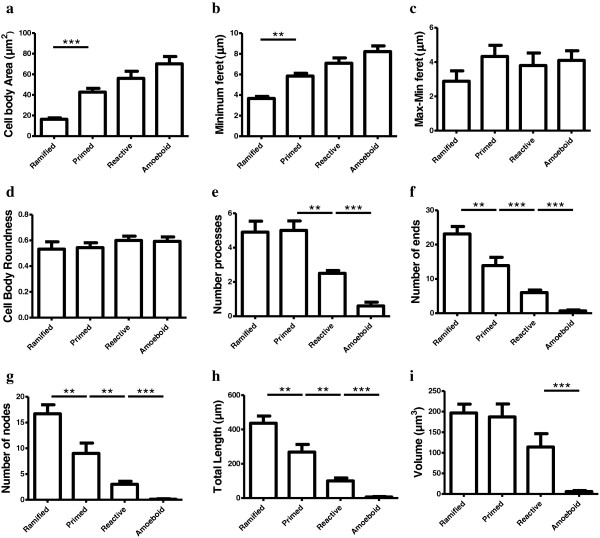
**Microglial phenotypes display significant differences in cell body and ramification patterns.** Compared to ramified, the primed phenotype displays a larger cell body area **(a)** as reflected by a significantly larger maximum (not shown) and minimum feret **(b)**. Despite this significant increase in area, the max-min feret **(c)** and the roundness **(d)** of the cell body did not statistically differ between these or any of the other phenotypes. The cell body morphology of reactive, primed and amoeboid microglia appears comparable at all points **(a-d)**. Ramified and primed microglia both extend similar numbers of primary processes **(e)**, however, primed microglia hold fewer ends **(f)** and nodes, **(g)** as well as significantly shorter processes **(h)** with no significant changes in volume **(i)** compared to ramified microglia. Reactive microglia extend a significant decrease of primary **(e)** and higher-order branches **(f-g)**, as well as shorter processes than primed microglia **(h)** and amoeboid microglia present almost an absence of overall processes **(e-g)** that were thus, of significantly reduced length **(h)** and volume **(i)** compared to reactive microglia; *P <0.01, **P <0.001, ***P <0.0001.

Similar to gray matter, all microglial phenotypes were observed in the white matter. Again, the majority of IBA1-IR cells displayed extensively branched processes. However, contrary to their grey matter counterparts, these cells had an oblong cell body from which emerged a bipolar arborization. Furthermore, ramified microglia in the white matter appeared aligned to myelinated fiber tracts (Figure 1b). From the total number of cells quantified in the white matter (n = 1,534), ramified microglia accounted for 43% of the total number of IBA1-IR cells, compared to 27% for the primed phenotype. The primed phenotype could be distinguished by the presence of a wider cell body and a significantly larger cell body area than ramified microglia (*t*_Welch__(11)_ 6.89, *P* <0.0001, Figure 5a); a feature that was reflected by a significantly larger maximum (*U*_(18)_ 10.50, *P* = 0.0032) and minimum feret (*t*_(18)_ 6.65, *P* <0.0001, Figure 5b). Yet, the roundness (Figure 5d) and the max-min feret of the cell body did not statistically differ between primed and resting microglia (Figure 5c). Ramified and primed microglia both presented a similarly high number of primary processes (Figure 5e). However, primed microglia presented fewer higher-order branches, as reflected by numbers of ends and nodes (*U*_(18)_ ≤13.00, *P* ≤0.0057, Figure 5f and 5 g), as well as significantly shorter processes compared to ramified microglia (*U*_(18)_ 13.00, *P* = 0.0039, Figure 5h).

As measured in the grey matter, reactive and amoeboid phenotypes in the white matter each represented slightly more than 11% and 19%, respectively, of the total number of IBA1-IR cells in dACC white matter. The cell body morphology of reactive and primed microglia was comparable in all points (area, maximum feret, minimum feret, max-min feret and roundness, Figure 5). However, reactive microglia presented a readily observable decrease of primary (*U*_(18)_ 13.0, *P* = 0.0030, Figure 5e) and higher order branches (*U*_(18)_ ≤9.50, *P* ≤0.0054, Figure 5f and 5 g), as well as shorter processes (*t*_Welch (11)_ 3.66, *P* = 0.0037, Figure 5h) than primed microglia. The cell body morphology of amoeboid microglia was measured to be statistically comparable to that of reactive microglia (Figure 5). However, the former showed a near absence of overall processes (primary and higher order) (*U*_(18)_ ≤6.50, *P* ≤0.0005, Figure 5) that were thus of significantly reduced length and volume compared to reactive microglia (*U*_(18)_ ≤6.0, *P* ≤0.0003, Figure 5h and 5i). As in gray matter, a few IBA1-IR cells were observed to be closely associated with large blood vessels in the white matter. All results for white matter cells are summarized in Table 2.

**Table 2:** Quantitative features of IBA1-IR phenotypes in human dACC white matter

**Cell body**					
	**Area (μm2)**	**Roundness**	**Min feret (μm)**	**Max feret (μm)**	**Max-min feret (μm)**
**Ramified**	16.40 ± 1.2	0.5 ± 0.0	3.6 ± 0.19	6.5 ± 0.5	2.88 ± 0.6
Range	9.11 to 24.4	0.2 to 0.8	2. to 4.8	4.8 to 9.9	1.7 to 5.6
**Primed**	42.74 ± 3.6	0.5 ± 0.0	5.8 ± 0.26	10.1 ±0.7	4.33 ± 0.6
Range	25.27 to 64.2	0.4 to 0.7	4.7 to 7.3	6.8 to 14.3	2.1 to 7.0
**Reactive**	59.6 ± 6.4	0.6 ± 0.0	7.4 ± 0.5	11.0 ± 08	3.5 ± 0.7
Range	18.3 to 89.9	0.4 to 0.7	3.9 to 9.0	6.0 to16.0	2.1 to 7
**Amoeboid**	70.2 ± 7.0	0.5 ± 0.0	8.2 ± 0.5	12.3 ± 0.7	4.1 ± 0.5
Range	37.3 to 111.9	0.4 to 0.7	5.8 to 11.2	8.7 to 16.2	2.9 to 4.9
**Processes**					
	**Primary processes**	**Ends**	**Nodes**	**Total length (μm)**	**Volume (μm**^**3**^**)**
**Ramified**	4.9 ± 0.6	23.1 ± 2.1	16.7 ± 1.7	436.0 ± 42.4	196.7 ± 21.4
Range	2.0 to 9.0	16.0 to 41.0	11.0 to 30.0	290.3 to 761.7	124.8 to 193.1
**Primed**	5.0 ± 0.5	13.9 ± 2.3	9 ± 2.02	268.2 ± 44.3	187.2 ± 31.3
Range	2.0 to 8.0	7.0 to 33.0	3.0 to 25.0	123.6 to 594.5	62.1 to 398.2
**Reactive**	2.8 ± 0.2	6.3 ± 0.7	3.2 ± 0.6	107.7 ± 17.0	125.2 ± 30.5
Range	2.0 to 3.0	3.0 to 9.0	0.0 to 6.0	25.9 to 187.8	10.8 to 278.9
**Amoeboid**	0.6 ± 0.2	0.7 ± 0.2	0.1 ± 0.1	6.8 ± 2.5	5.9 ± 2.5
Range	0.0 to 2.0	0.0 to 2.0	0.0 to 1.0	0.0 to 19.5	0.0 to 20.2

Results are presented as mean ± standard error of the mean and range.

### Microglial phenotypes in mouse cingulate cortex

**Figure 6:**
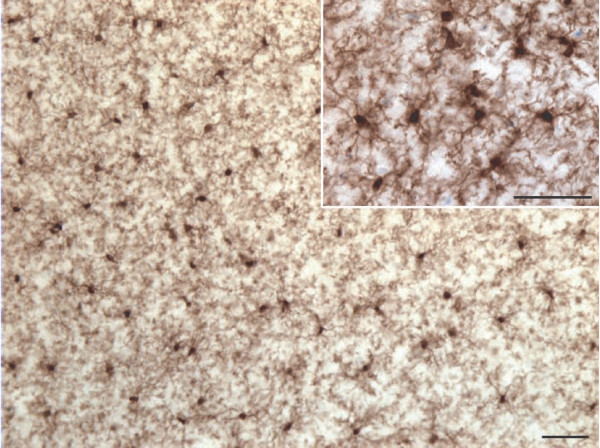
**IBA1-IR cells in the cingulate cortex of a young adult mouse (1.5 months old) display cell bodies of heterogeneous shapes and sizes, and highly ramified processes with overlapping domains.** Scale bars: 50 μm.

As mentioned above, IBA1-IR cells present in the mouse cingulate cortex were generally highly branched, and their cell bodies were evenly distributed in the tissue. The densely packed arborizations extended by IBA1-IR cells in 3-month-old mice were organized into distinct and non-overlapping domains. Although the morphology of IBA-IR cells was similar in the younger adult mouse (1.5 months old), highly branched arborizations of neighboring cells were often found to overlap at their extremities, suggesting a regional organization that is not yet fully mature at that age (Figure 6). Reactive and amoeboid-like morphologies were rarely observed in adult mice. As opposed to what was observed in human tissues, highly ramified IBA1-IR cells in mice presented heterogeneous cell bodies, with varying shapes and sizes (Figure 6). A proportion of these cells could also have been of the primed phenotype, but could not be distinguished from the ramified phenotype because of the inconsistent cell body morphology of highly branched cells in rodents. In consequence, if using the same criteria as those used for human samples, it remains possible that a subset of these ramified microglia observed in young adult mice were in fact of the primed phenotype. In the white matter, the cell body of ramified microglia were more uniform in terms of shape and size, and their processes displayed a bipolar organization that was aligned in a non-overlapping fashion to white matter tracts.

**Figure 7:**
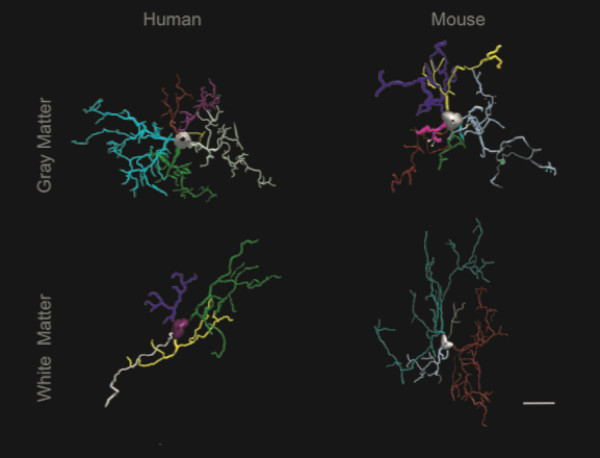
**Representative reconstructions of gray matter (top row) and white matter (bottom row) ramified microglia in human dACC (left column) and mouse cingulate cortex (right column).** Scale bar: 10 μm.

Comparisons of reconstructed mouse and human ramified IBA1-IR cells showed that despite a significant increase in cell body area (*t*_(18)_ 4.02, *P* = 0.0008) and high-order branches (ends and nodes *U*_(18)_ 5.0, *P* ≤0.0008) in the mouse white matter, the surface area and volume of processes in the gray and white matter were statistically similar. In the gray matter, the heterogeneity in cell body shape was reflected by a significant decreased roundness (*t*_Welch (11)_ 4.92, *P* = 0.0005) and max-min feret (*t*_Welch (9)_ 4.93, *P* = 0.0008) in the mouse microglial ramified phenotype. All other parameters were statistically comparable between groups (Figure 7).

### Effects of postmortem interval on microglial phenotypes in mouse cingulate cortex

**Figure 8:**
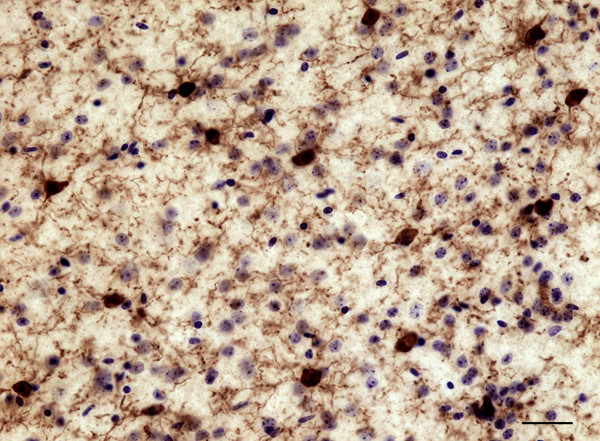
**IBA1-IR cells in the non-perfused cingulate cortex of a of young adult mouse (3 months old) following a PMI of 43 h (11 h at room temperature and 32 h at 4˚C).** The great majority of IBA1-IR cells observed remained ramified, and did not display noticeable signs of degradation. Sections were counterstained with cresyl violet. Scale bar: 25 μm.

As in brains harvested and fixed at time of death, brains fixed after a PMI of 43 h displayed ramified IBA1-IR microglia as the predominant phenotype in both gray and white matter. Furthermore, these cells displayed no noticeable degradation of their processes (Figure 8). Some reactive and amoeboid IBA1-IR cells were also observed following this PMI (approximately 6% and 4%, respectively). These scarce cells were found in both cortical gray and white matter, but rarely in association with any particular structure, such as blood vessels.

## Discussion

In this study, we describe the morphometric features of IBA1-IR microglial cells in adult human gray and white matter dACC from individuals having died without inflammatory, neurological, or psychiatric illness. In the gray matter, microglial cells were abundant and evenly distributed, with neither overlapping domains nor obvious differences between cortical layers. This uniform distribution is hypothesized to allow microglial cells to efficiently and constantly survey the microenvironment with their highly motile processes [45]. This dynamic phenomenon has been implicated in many physiological activities, such as maintaining the viability of synaptic contacts [1,46]. In white matter, IBA1-IR microglial cells were aligned with myelinated tracts, which generally conferred an oblong shape to their cell bodies. Cells were more abundant in proximity to the gray matter compared to deeper white-matter regions, which displayed much more discrete staining.

Based on clear morphological differences described previously in humans and rodents [14,22,43], we confirm the existence of four major microglial phenotypes in adult human cortical gray and white matter. In the gray matter, ramified microglia were characterized by very extensive branching patterns and small spherical cell bodies. Primed microglia presented similar arborization patterns compared to resting microglia, but displayed an overall increase in cell body area as well as a decrease in roundness. Reactive microglia featured an amoeboid-like cell body with few ramified processes, whereas amoeboid microglia extended either a single unramified process or no processes at all. It has to be acknowledged here that this nomenclature, which is derived from previous morpho-functional studies, may not always accurately reflect the functional states of microglial cells, or whether a cell is transitioning towards increased activation or reverting back to a ramified morphology. Furthermore, it is impossible to determine in postmortem brain tissues, the proportion of IBA1-IR cells that are resident microglia versus infiltrated monocytes that have differentiated [47].

Significant numbers of IBA1-IR cells displaying each of the four morphological phenotypes were observed in both cortical compartments of all subjects. Estimates showed that the majority of IBA1-IR microglia in the gray matter were of the primed phenotype (34%), followed by the reactive (32%), amoeboid (18%) and ramified (16%) phenotypes. The proportions were different in the white matter, with ramified cells representing the majority (43%), followed by the primed (27%), amoeboid (18%) and reactive (12%) phenotypes. Interestingly, amoeboid cells consistently accounted for nearly a fifth of the total number of IBA1-IR cells in the dACC. These results contrast dramatically with those observed in the cingulate cortex of young adult mice, in which IBA1-IR cells were overwhelmingly highly branched, and most likely of the ramified phenotype. Other microglial phenotypes were very rarely observed in mice. In order to determine whether the presence of different microglial phenotypes in human tissues may have resulted at least partly from postmortem factors, we recreated similar PMI conditions in the mouse. Although we observed a slight increase in reactive IBA1-IR cells following a PMI of 43 hours compared to immediate harvesting of the brain following sacrifice, the great majority (≥90%) of microglial cells remained of the ramified phenotype.

These observations strongly suggest that the proportions and distributions of the different microglial phenotypes in human dACC were mostly present at time of death in individuals who did not suffer from inflammatory, neurological or psychiatric illness. This is further supported by previous reports demonstrating that microglial morphological changes require ATP [48], oxygen and glucose supply [49], all of which cease shortly after death, leaving little opportunity for microglial cells to undergo phenotypic changes, let alone retract their processes and adopt an amoeboid phenotype [48].

Another noticeable difference between human and mouse microglia relates to the shape of cell bodies. Whereas human microglia displayed characteristic round (ramified phenotype) or amoeboid-shaped (primed, reactive, amoeboid phenotypes) cell bodies, mouse microglia displayed highly heterogeneous cell-body shapes that prevented the distinction of ramified versus primed phenotypes. Despite this difference, the size and general morphology of microglia were found to be highly similar between species. Microglia are thus different from astrocytes, another glial cell type, which are several-fold larger as well as much more diverse and complex in the human than in the mouse cerebral cortex [50]. This morphological similarity between species may be related to the different developmental origin of microglia, and to highly conserved roles in mammalian evolution.

The observed variability of microglial distributions between human subjects appears to be due to variations in inter-cell spacing and cell densities within each sample. In rodents, it has been shown that age could influence the spacing between neighboring microglia [42]. Similarly, stress is another factor that can influence microglial morphology and distribution [51]. These (and probably other) factors may have contributed to the inter-individual variations in microglial distribution in human brain samples.

In conclusion, this study is the first to provide morphometric characterization of microglial morphology in humans. Interestingly, we found that the general morphological features of human microglia in the dACC were highly similar to those displayed by microglia in the mouse cingulate cortex. Thus, the study of microglial anatomy in rodent models seems more appropriate than that of other glial subtypes such as astrocytes, which display highly distinctive characteristics in humans. Our results indicate that four major microglial phenotypes co-exist in adult human cortical tissues. Given that the average age of the subjects analyzed in this study was 48 years, it is possible that this phenotypic distribution is a normal consequence of the aging process. This hypothesis could be tested in future studies of postmortem cortical samples from adolescents or young adults. Finally, the quantified data generated in this study will be instrumental in future studies examining the implication of microglia in various conditions and illnesses thought to arise, at least in part, from abnormal immune activity in the brain. As suggested previously, comparing microglial phenotypic ratios in well-characterized brain samples may be particularly instructive to understand the state of inflammatory processes in a given brain circuitry [52]. Likewise, this approach would allow the precision required to identify subtle imbalances in microglial phenotypic distributions that may characterize illnesses associated with a mild inflammatory component.

## Abbreviations

IBA1: ionized calcium binding adaptor molecule 1; dACC: dorsal anterior cingulate cortex; CNS: central nervous system; PBS: phosphate-buffered saline; PMI: postmortem interval; PFA: paraformaldehyde; IR: immunoreactive; TBS: Tris-buffered saline.

## Competing interests

All the authors declare that they have no competing interests.

## Authors’ contributions

SGTP conceived and designed the study, processed the human tissue, carried out most experiments, analyzed and interpreted the data, and drafted the manuscript. SC was involved in tissue processing, carried out some experiments and acquired part of the data. AR was involved in tissue processing and immunohistochemical experiments. GDB processed animal tissues and contributed to data analysis. CC participated in the design of the study and contributed to data analysis. GT and BG participated in the design of the study and in the interpretation of the data. NM conceived and designed this study, supported and coordinated experiments, interpreted the data and drafted the manuscript. All authors read and approved the final version of the manuscript.
